# Forecasting of Cereal Yields in a Semi-arid Area Using the Simple Algorithm for Yield Estimation (SAFY) Agro-Meteorological Model Combined with Optical SPOT/HRV Images

**DOI:** 10.3390/s18072138

**Published:** 2018-07-03

**Authors:** Aicha Chahbi Bellakanji, Mehrez Zribi, Zohra Lili-Chabaane, Bernard Mougenot

**Affiliations:** 1 LR17AGR01 (LR GREEN-TEAM)/INAT, University Carthage, Avenue de la République, P.O. Box 77, Carthage, Tunis 1054, Tunisia; chehbi.aicha@gmail.com (A.C.B.); zohra.lili.chabaane@gmail.com (Z.L.-C.); 2 ISET_Nabeul, Campus Universitaire Mrezgua, Nabeul 8000, Tunisie; 3 CESBIO (CNRS/UPS/IRD/CNES), 18 Avenue Edouard Belin, 31401 Toulouse CEDEX 9, France; Bernard.mougenot@ird.fr

**Keywords:** yield, cereal, SAFY, optical remote sensing, SPOT/HRV

## Abstract

In semi-arid areas characterized by frequent drought events, there is often a strong need for an operational grain yield forecasting system, to help decision-makers with the planning of annual imports. However, monitoring the crop canopy and production capacity of plants, especially for cereals, can be challenging. In this paper, a new approach to yield estimation by combining data from the Simple Algorithm for Yield estimation (SAFY) agro-meteorological model with optical SPOT/ High Visible Resolution (HRV) satellite data is proposed. Grain yields are then statistically estimated as a function of Leaf Area Index (LAI) during the maximum growth period between 25 March and 5 April. The LAI is retrieved from the SAFY model, and calibrated using SPOT/HRV data. This study is based on the analysis of a rich database, which was acquired over a period of two years (2010–2011, 2012–2013) at the Merguellil site in central Tunisia (North Africa) from more than 60 test fields and 20 optical satellite SPOT/HRV images. The validation and calibration of this methodology is presented, on the basis of two subsets of observations derived from the experimental database. Finally, an inversion technique is applied to estimate the overall yield of the entire studied site.

## 1. Introduction

In arid and semi-arid areas, population growth, urbanization, food security and climate change have an impact on agriculture in general and in particular on cereal production. Since the 1980s North African countries have experienced significant increases in their cereal imports. Morocco, Algeria and Tunisia account for eight per cent of world cereal imports, while they account for only one percent of the world’s total population [1]. Therefore, to improve food security in arid countries, crop canopy monitoring, yield forecasting cereals and the planning of annual imports are needed [2]. For years, the scientific community has demonstrated the potential of remote sensing for the spatio-temporal monitoring of the soil properties, vegetation cover and grain yield of cereals [3,4,5,6].

Three main groups of modeling techniques used for rough scale yield estimations can be distinguished: (i) qualitative yield forecasting, and two types of quantitative yield forecasting: (ii) regression models and (iii) the combined use of models of cultivated fields and remote sensing data.

Qualitative crop monitoring methods are based on remote sensing observations, and are applied at national and regional levels. They provide qualitative, but not quantitative descriptions, and are generally based on a projection of the actual crop status observed during previous seasons. This approach is used by the Food and Agriculture Organization of the United Nations (FAO) for countries in Africa, Asia and South America [7], the Famine Early Warning System (FEWS) NET, the United States Agency for International Development (USAID), and the Monitoring Agriculture with Remote Sensing (MARS) program of the European Commission [8].

On the other hand, regression models can provide quantitative evaluations, based on the interpretation of a time series of satellite images and a land use map. In addition, the latter type of model must necessarily be calibrated using appropriate reference information, such as agricultural statistics and crop yield measurements. Regression-model based forecasting techniques can be subdivided into two types: those which use remote sensing data only (empirical regression models); and mixed techniques, which include additional bio-climatic predictor variables. Optical imaging techniques (from visible to thermal: 0.4 μm to 12.5 μm) are suitable for determining parameters characterizing vegetation and that by combining reflectance in different wavelength bands, in the form of vegetation indices. The vegetation is different from the other types of surface because it absorbs wavelengths in the red of solar radiation, and reflects in the near-infrared. The index most frequently used is the Normalized Difference Vegetation Index (NDVI). This index is considered as a reliable indicator of green vegetation and land-cover variations since its temporal variations are closely linked to the presence of vegetation. Other studies have shown the very high performance of the Enhanced Vegetation Index (EVI) for monitoring and assessing spatial and temporal variations in vegetation amount and condition [9,10].

Many models use the NDVI to estimate crop yields. Examples of this type of model include the model of [11] used at state level in Kansas (USA) and in the Ukraine; the model of [12], which was applied to three countries (the United States, Ukraine and China) between 2001 and 2012; and the method proposed by [13], which was applied in a semi-arid catchment in central Tunisia. These types of model rely mainly on statistical analysis of the vegetation index “NDVI”, which is commonly used in remote sensing, and the measured yields. In Chahbi et al. [13], the early estimation of cereal yield is based on a statistical analysis between the vegetation index NDVI and the yields measured on the test plots. An estimation of cereal yield is possible from mid-March, when the cereal flowering phase is achieved. The validation of the remote sensing estimation shows that this approach is robust, with a high correlation coefficient R^2^ generally greater than 0.6. These estimations show errors equal to 8.5 qx/ha and 11.6 qx/ha, respectively, for grain and straw yields. In this study, yield maps are proposed.

Although regression models are simple to apply, efficient, and more accurate than qualitative methods, it is difficult to extend a model that has been calibrated for a pilot area to other regions or other scales.

A third technique can be used to forecast crop yields, based on the use of growth models: Soil Vegetation Atmosphere (SVAT) modeling or agro-meteorological modeling [13,14,15,16,17]. Reference [13] proposed the application of the SAFY semi-empirical growth model, developed to simulate the dynamics of the leaf area index (LAI) and grain yields, at the field scale. Although this model is able to reproduce temporal variations of LAI for all fields, their yields are under-estimated. In practice, the statistical analysis proposed by [13] does not identify a fixed date for the relationship between vegetation indices and yields, which can lead to additional errors in the analysis, from one year to the next. To eliminate this problem, we propose an approach based on the integration of measurement. The aim of the present study is to evaluate the feasibility of a hybrid approach, combining the SAFY agro-meteorological model with remotely sensed images, at times corresponding to the maximum growth of cereals. 

Section 2 is devoted to the description of the study site, the satellite data and the ground measurement. A description of the SAFY model is presented in Section 3. Furthermore, the methodology used to estimate the dynamics and yields of cereals is described. Section 4 is dedicated to present the results and discussions of this method. The conclusions are presented in Section 5.

## 2. Experimental Database

### 2.1. Study Area

**Figure 1:**
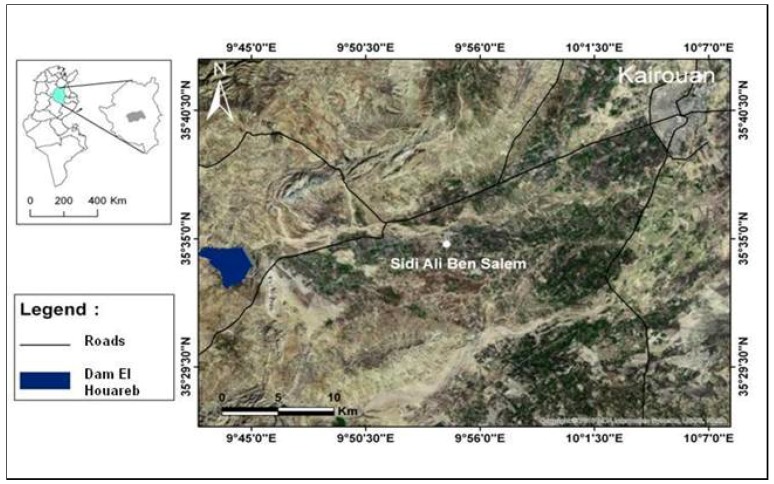
Illustration of the studied site.

The region of Kairouan is located in the eastern zone of the Tunisian Atlas, between central Tunisia and eastern Tunisia: latitude 9°30′ E–10°15′ E and longitude 35° N–35°45′ N (Figure 1). This area is characterized by a semi-arid climate. In central Tunisia, the rainy season extends from October to May, in summer, rain is almost absent. In the Kairouan region, the average annual rainfall is 400 mm per year on the heights and 300 mm per year in the plain [18]. The evapotranspiration (Penman) in this region is close to 1600 mm.

In the Kairouan plain, the main economic activity is agriculture. The area is dominated by farming, arboriculture (irrigated and non-irrigated), cereals (wheat and barley) with a notable increase in polyculture. In the recent years, intensive agriculture has been encouraged by the government [19].

### 2.2. Satellite Data

For North Africa, farms are for the most part small plots, due to inheritance processes that have favored the fragmentation of land [20]. Consequently, the study of agricultural surfaces by remote sensing requires the use of satellite images with a high spatial resolution such us SPOT Image (spatial resolution is equal to 10 m). Fifteen images acquired by the SPOT 5 and SPOT4 satellites were analyzed (Table 1).

These images were acquired with a repeat time of approximately 21 days and a high spatial resolution, equal to 10 m and 20 m for SPOT5 and SPOT4, respectively. Each image has four bands: green, red, near infrared (PIR) and mid-infrared MIR. In Table 2 we have the bands characteristics of the two satellites SPOT4 and SPOT5. 

Radiometric and atmospheric corrections were then applied in order to estimate the reflectance of the vegetation canopy. For atmospheric correction, the Second Simulation of Satellite Signals in the Solar Spectrum (SMAC 6s) model [21,22,23] was used. This model is based on an improvement of the 5s model developed by [24]. The SMAC 6s model takes into account the atmospheric parameters (ozone content, optical thickness, etc.), the geometric parameters of shooting (incidence angle, the zenith angle of the sun and the satellite, etc.). In order to limit the effect of incidence angle on measured reflectance, all acquired images were taken with an incidence angle lower than 19°.

### 2.3. Ground Measurements

**Figure 2:**
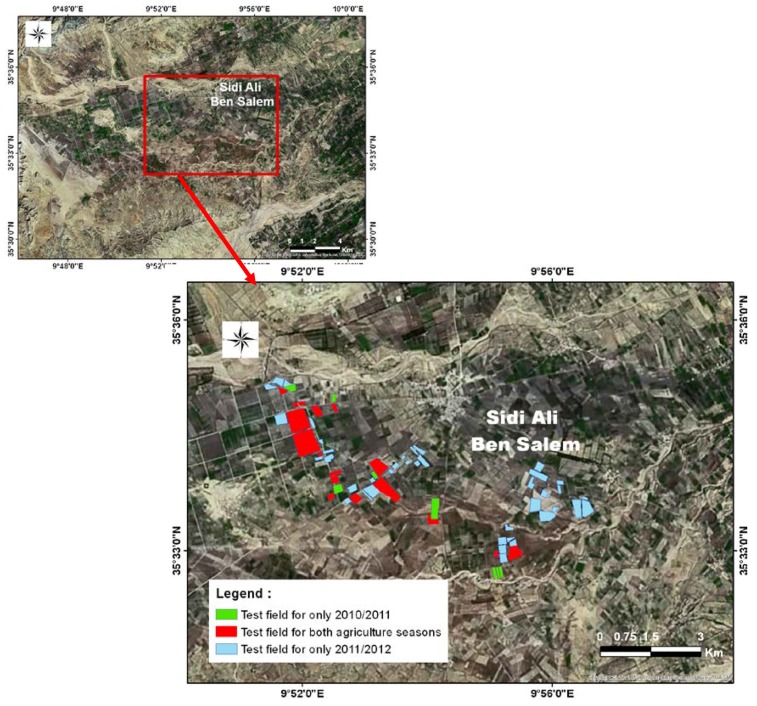
Color-coded view of the test fields.

Various ground measurements were carried out during two agricultural years: 2010/2011 and 2011/2012, covering 27 test plots in the first year and 55 plots in the second year (Figure 2). The test plots were selected in such a way as to ensure a combination of wheat and barley fields that were both rain-fed and irrigated. 

**Figure 3:**
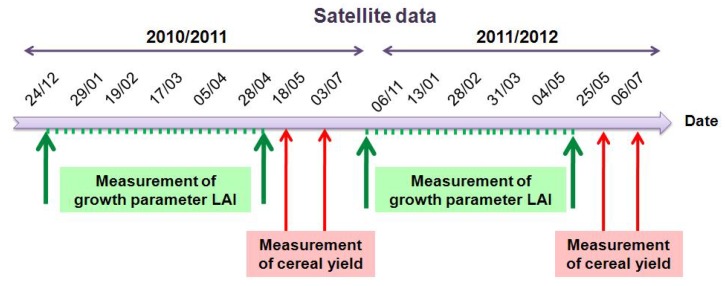
Overview of the satellite and ground measurements.

In order to be sure of the quality of statistical analysis, only fields with more than one hectare were considered in this selection. The in situ measurements included mainly: water content, crop height, Leaf Area Index (LAI) of the vegetation, and cereal yield at the end of the season. In Figure 3, we present an overview of the satellite and ground measurements during the two agricultural years.

#### 2.3.1. Leaf Area Index

The LAI is defined as the total one-sided area of leaf tissue per unit of ground surface area. According to this definition, the LAI is a dimensionless quantity characterizing the canopy of an ecosystem. The LAI measurements were made on all test plots during different stages of vegetative development of the cereals, with a repeatability of two to three weeks during the agricultural season between December and April. In this study, hemispherical digital photography was used to measure the LAI. Thirty LAI estimations were made in two 20 × 20 m square zones inside each field. The LAI was derived from hemispherical digital photography based on analysis of the canopy gap fraction [16]. The proposed approach was validated by destructive measurement campaign carried out in the past over the studied site [13]. Despite the good agreement between the two approaches, a decrease in accuracy exists for non-destructive approaches for dense vegetation covers.

#### 2.3.2. Cereal Yield

The cultivated area, production, and crop yield data are essential elements for agricultural statistics. The average yield is the average quantity produced per unit of cultivated area. Several different methods can be used to estimate or measure cereal yields. In the present study, a “many small frame” sampling technique was implemented [25], using a 75 × 75 cm frame. Ten samples were taken along the two diagonals of each field, and the cereal yields were then measured: the number of stalks, the weight of the grain, and the weight of the straw were recorded. From these measurements, the average grain and straw yield was determined for each field. For both agricultural years, the grain yields ranged from 11 qx/ha to 85 qx/ha, with an average value of 32 qx/ha, whereas the straws yields ranged from 8 qx/ha to 70 qx/ha, with an average value of 28 qx/ha.

### 2.4. Classification of Satellite Images over the Kairouan Plain 

#### 2.4.1. Land Use Map

**Figure 4:**
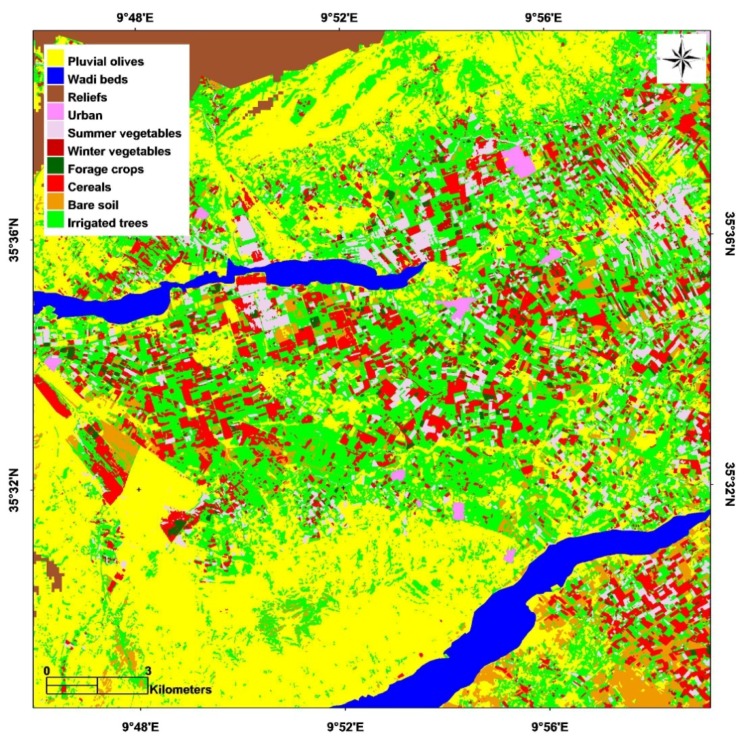
Land use map for the 2010–2011 agricultural season.

Land use identification (Figure 4) is an essential step in the process of remotely mapping the yield of cereal fields. In the present context, this involved the production of an annual land use map of the Kairouan plain, based on a suitably adapted classification system, taking into account possible changes from one year to the next. A decision-tree type of classification was used to transpose the optical images into land use maps, for the agricultural seasons of 2010/2011 and 2011/2012. This method is based on the use of vegetation class detection thresholds, and in the present case the variable used was the Normalized Difference Vegetation Index (NDVI) [13,26]. In our study, a preliminary study of NDVI profiles generated using SPOT images was performed in order to determine the thresholds with which the separation between cultures is possible or not. For all classes, thresholds were considered from one image or from a difference between two images. Test fields were used during the learning phase in order to identify empirical NDVI thresholds allowing the different classes to be distinguished. For example, for summer vegetables, an empirical NDVI threshold was applied to the image acquired in July (NDVI > 0.3).

In order to assess the cereal classes (wheat or barley, irrigated or non-irrigated), satellite data recorded during March or April, corresponding to the period of maximum cereal growth, was analyzed. Pluvial olive groves cover 44% of the study area, whereas cereals corresponded to 8% of the total classified area in 2010/2011, and 22% of this area in 2011/2012. These remotely sensed classifications were validated through the use of qualitative observations over more than 100 fields in the studied site, characterized by different types of land use, revealing an overall accuracy of approximately 80%. Once the land use map has been established, it can be used to define a cereal mask for the two agricultural seasons under study.

#### 2.4.2. Classification of Irrigated and Non-Irrigated Cereals 

In this section, we focus on the definition of a cereal mask used to identify two different types of cereals: irrigated and non-irrigated. For this, an object-oriented classification technique was used, in which the classification takes place in two stages: segmentation of the image into objects, followed by classification of this segmentation. The object-oriented approach operates on adjacent pixel groups, and makes use of various spectral and spatial homogeneity criteria (spectral value, shape, texture, etc.) in order to discern between the objects to be classified [27]. The decision rules used for such an operation include not only the spectral, but also the spatial parameters. This process is inspired from the multi-resolution segmentation concept proposed in the Orfeo Toolbox OTB Monteverdi software [28].

As cereal vegetation growth peaks in the month of March, the Spot images used in this study were recorded on 17 March 2011 for the 2010/2011 agricultural season, and 31 March 2012 for the 2011/2012 season, and the cereal masks corresponding to these images were applied. The segmentation of these images was carried out using the Mean-shift Clustering principle [29,30,31,32,33]. 

**Figure 5:**
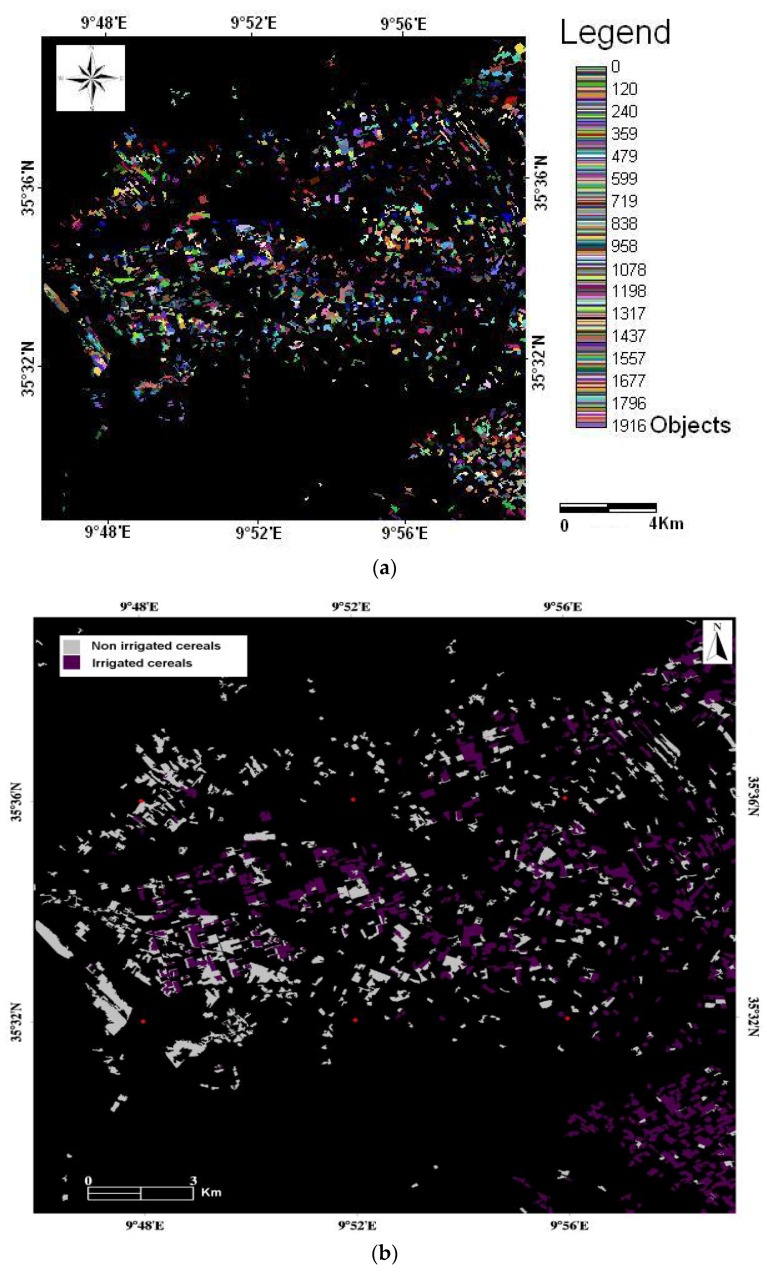
(**a**) Segmented image produced for the 2010/2011 agricultural season; (**b**) Land-use map of irrigated and non-irrigated cereals for the 2010/2011 agricultural season.

Figure 5a shows a segmented image of the Kairouan plain for the 2010/2011 agricultural season. Once the image has been segmented into spatial objects, which in reality correspond to homogeneous cereal plots, they are classified according to agricultural practice: non-irrigated or irrigated. This classification is based on vegetation index (NDVI, TSAVI…). Plots chosen for the validation of classification come from field observations in the study area. The choice of these plots was made in order to have a mixture between wheat and barley and which are conducted either in rainfed or irrigated.

The segmented and classified image (Figure 5b) was validated using 55 known plots, 36 of which were irrigated and 19 were pluvial. The map corresponding to the 2010/2011 agricultural season has an overall accuracy of about 97.3%, and a precision Kappa coefficient equal to 94%. In the case of the 2011/2012 agricultural year, the overall coefficient is approximately 81%, and the precision Kappa coefficient is equal to 62%. These maps were thus considered to be valid for the study of cereal yields at the plot scale.

## 3. Methodology for the Estimation of Cereal Yields Using the SAFY Growth Model

### 3.1. SAFY Model Description

Over the last few decades, several techniques have been developed for the regional monitoring and spatialization of vegetation growth, based on the combined use of a model and remotely-sensed optical images. Among these approaches, the model of [34] provides the simulation of dry biomass production and the interception of light by vegetation. For the modeling of growth and crop yields, it can be advantageous to introduce the leaf area index (LAI), which is involved in the production of vegetative biomass and is a key variable in the functioning of crops. In this context, the Simple Algorithm for Yield Estimate (SAFY) model developed by [16] was used to take into account the main processes of cereal development and growth at the plot scale.

This model is based on the light-use efficiency theory of [34]. It provides a simulation of the increase in dry, above-ground phytomass. Also, it takes into account the influence of temperature and the dynamics of green leaves [16].

Each day of the vegetative period, the production of vegetative biomass is determined from the global radiation (Rg) in photo-synthetically active radiation via the climatic efficiency ε_C_. A Part of this radiation is absorbed by the vegetation cover through the light interception efficiency ε_I_. The conversion of the global radiation by ε_C_ and ε_I_ gives rise to photosynthetically active radiation absorbed by vegetation (APAR). This term will be converted to dry above-ground biomass (ΔDAM) through light-use efficiency (ELUE). Also, ΔDAM is affected by the daily average of air temperature (Ta) through the temperature-stress-function F_T_.

This conversion of global radiation to dry above-ground biomass is done by the following equations:

(1) $$\Delta \mathrm{DAM}=Rg\times \varepsilon_{C}\times \varepsilon_{I}\;.\mathrm{ELUE}\times F_{T}\left(T_{a}\right),$$

(2) $$\Delta \mathrm{DAM}=\mathrm{APAR}\times \mathrm{ELUE}\times F_{T}\left(T_{a}\right),$$

During the growth phase, part of the ΔDAM is allocated to leaf biomass. This fraction is calculated using the leaf partition function Pl which varies between 0 and 1. Thus, the daily production of leaf biomass (DAM × Pl) is converted into leaf area (ΔLAI^+^) via the specific leaf area SLA:

(3) $$\Delta \mathrm{LA}I^{+}=\Delta \mathrm{DAM}\times \mathrm{Pl}\times \mathrm{SLA}\;\;\mathrm{si}\;\mathrm{Pl}>0$$

The senescence of leaves starts when accumulated air temperature reaches a given threshold Stt. ΔLAI^−^ increases with thermal time (ΣTa) and is modulated by the Rs parameter which defines the rate of senescence:

(4) $$\Delta \mathrm{LA}I^{-}=\mathrm{LAI}\times \frac{\sum \mathrm{Ta}-\mathrm{Stt}}{\mathrm{Rs}}\;\;\mathrm{si}\;\mathrm{SMT}>\mathrm{Stt}$$

Thus, the LAI at day j is calculated from the LAI at day (j − 1), the term of increase of the leaf surface (ΔLAI^+^) and the term of senescence (ΔLAI^−^).

The SAFY model also simulates grain yield. The daily grain increase “ΔGY” is proportional to the dry above-ground biomass DAM and to the grain partition function P_y_. This is given by Equation (5):

(5) $$\Delta \mathrm{GY}=\mathrm{DAM}\times P_{y}$$

SAFY has a low level of complexity, which simplifies the optimization of unknown parameters (limited to not more than 14), using a small number of observations. From these parameters, a priori values were determined on the basis of various prior experimental studies [35,36]. However, three of these parameters (the day of plant emergence D_0_, the parameter ELUE, and the “sum of temperature for senescence” S_TT_) are strongly dependent on the ambient agro-environmental conditions. In the Table 3, we have the range of variation of these three parameters for the test plots.

### 3.2. Application of SAFY to Cereal Cycle Retrieval

The first step involves the calibration of the three aforementioned parameters: D_0_, ELUE, and S_TT_. This procedure involves the identification of an optimum parameter set from which, for each field, the SAFY simulation leads to the best reproduction of several observed variables.

These parameters are calibrated using an optimization algorithm based on the comparison between in situ values of LAI observed in the fields and those estimated with the SAFY model [13]. 

**Figure 6:**
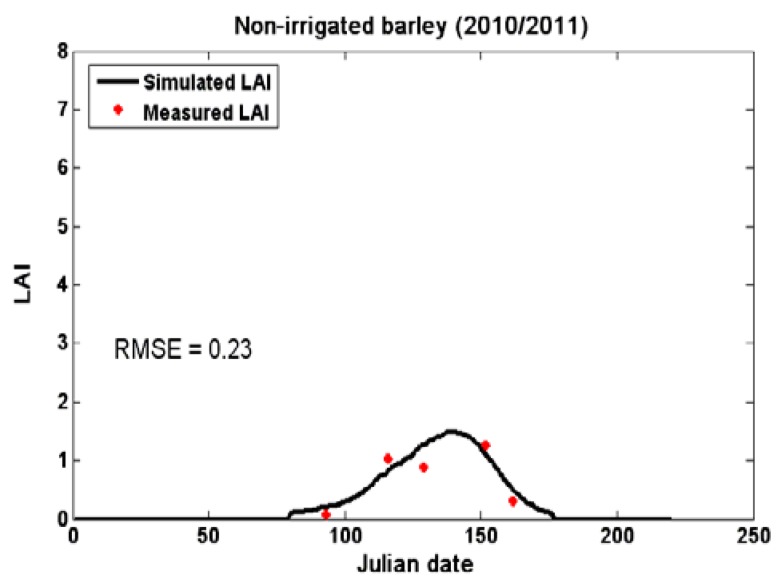

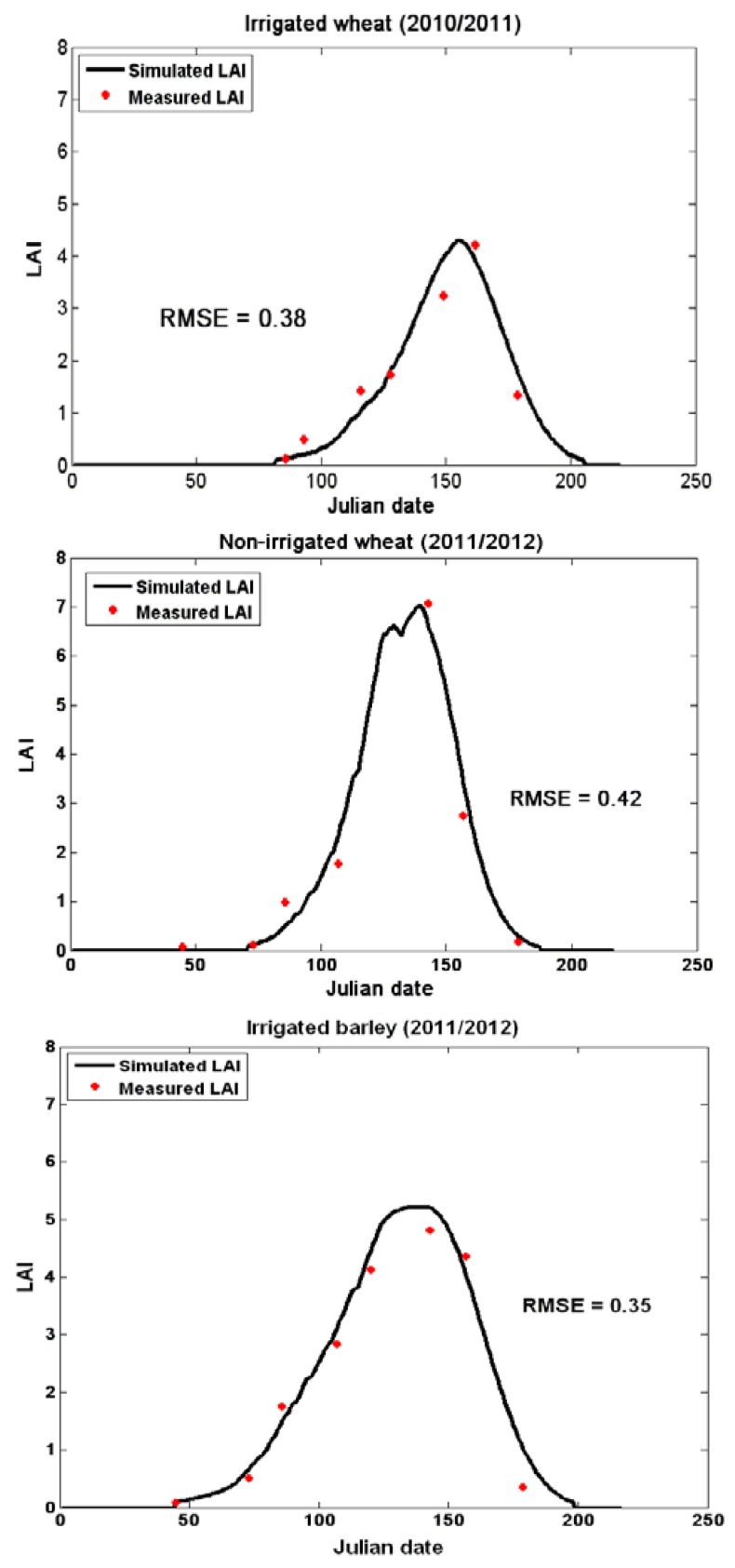
Values of observed (star) and simulated (line) LAI, estimated using the SAFY model for four test plots on the Kairouan plain during both agricultural seasons (1st Julian date = 1 November).

For each test plot, the optimized parameters (D_0_, ELUE, and S_TT_) lead to the lowest Root Mean Square Error (RMSE) between these two values (Figure 6). It can be obvious that the model correctly retrieves the dynamic of the LAI for test plots.

### 3.3. Proposed Approach

Although it has been shown that statistical empirical analysis, which associates the NDVI index during the period of maximum growth with the expected cereal yield, is highly accurate [13], it is not possible to predict a fixed annual date for this relationship. This can lead to additional errors in the analysis, from one year to another. In order to reduce this drawback, we propose an approach based on integral measurements of maximum growth recorded over a decade. The maximum value of cereal LAI at our site occurs towards the end of March, when the vegetation is flowering. Before this date, the maximum development of the vegetal cover is not yet reached for all the plots, it varies according to the date of sowing, the type of irrigation, the conduct of the fertilization, etc. After mid-April, the NDVI begins to fall, which may also lead to errors in yield estimation. 

**Figure 7:**
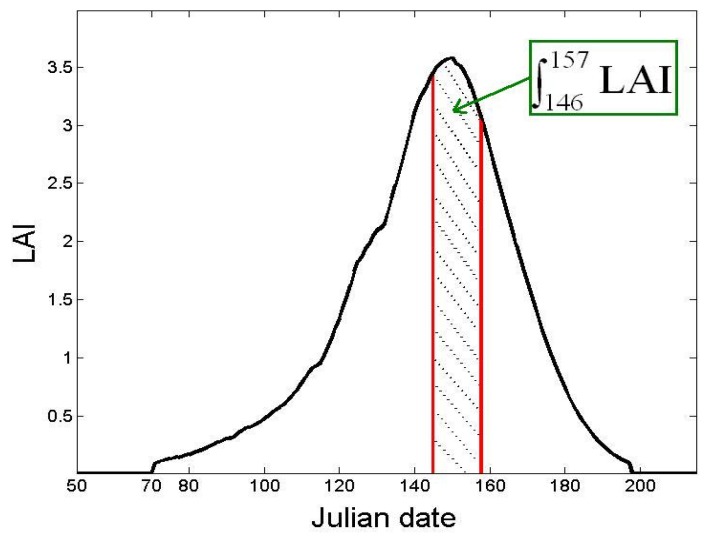
Principle of cereal yield estimations, based on the integral of the LAI over the period of maximum canopy development (A_LAI_).

The grain yield is thus expressed as a function of LAI, during the cereal development period between 25 March and 5 April, which corresponds to the range between the 146th and 157th Julian Days. We note this parameter A_LAI_ (Figure 7). In the initial SAFY model, the yield is expressed as a function of the total dry aerial phytomass (including, among others, the maximum LAI). Application of the SAFY model to the study of the Kairouan plain raised various questions concerning the model’s limitations in terms of yield estimation. It was thus proposed to improve these estimations by including the LAI integral (A_LAI_) in the grain yield calculation. Since the SAFY model accurately extracts the variation of LAI along the vegetative cycle, the estimation of A_LAI_ will be established by this model for each plot (segment). Consequently, the phenological development and the sowing date (D_0_) were taken into consideration:

(6) $$\mathrm{GY}=f\left(\int_{146}^{157}\mathrm{LAI}\right)$$

## 4. Results and Discussions 

### 4.1. Estimating the Yields of All Cereals

For this part, our database set was divided into three disjoint folds of the same size. In these sequential folds, the first was used for validation procedures, and the two remaining folds were used for assessing the yield model. As in the case of the “repeated holdout” method, the overall accuracy is given by the average of the values obtained from all runs.

**Figure 8:**
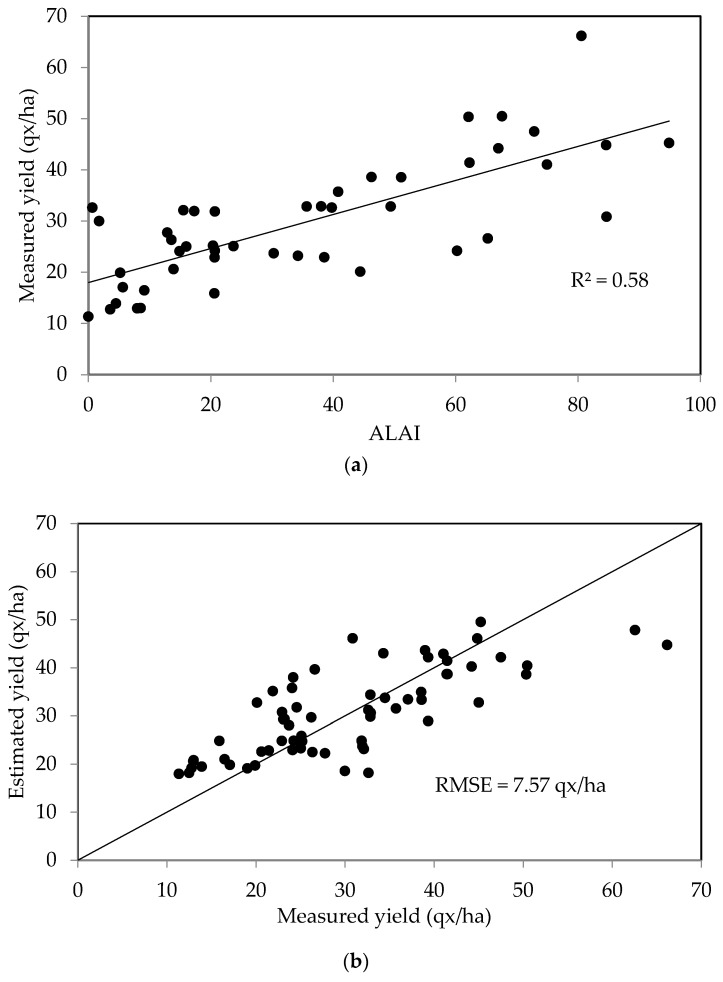
(**a**) Relationship between measured cereal grain yields and A_LAI_; (**b**) Measured grain yields compared with yield estimates, computed using the maximum growth period LAI, for two test plots and two crop years.

The method described in the previous section was initially applied to the first data set, regardless of the variety of crops and cultivation techniques used (wheat, barley, irrigated, non-irrigated). Figure 8a shows a plot of the raw data, expressing measured grain yields as a function of A_LAI_, together with the corresponding linear regression line. The two confidence intervals are also shown: the nearest interval to the curve is around the mean of the estimator and the farthest is the interval around the point estimate). The two variables are correlated (R^2^ = 0.58):

(7) $$GY_{\mathrm{qx}/\mathrm{ha}}=0.33A_{\mathrm{LAI}}+17.97$$

Figure 8b shows a plot of the measured yields as a function of the values estimated using the maximum LAI area for the second data set. These two parameters are found to be well correlated, with the RMSE equal to 7.57 quintals/ha.

### 4.2. Wheat and Barley Yield Estimations

**Figure 9:**
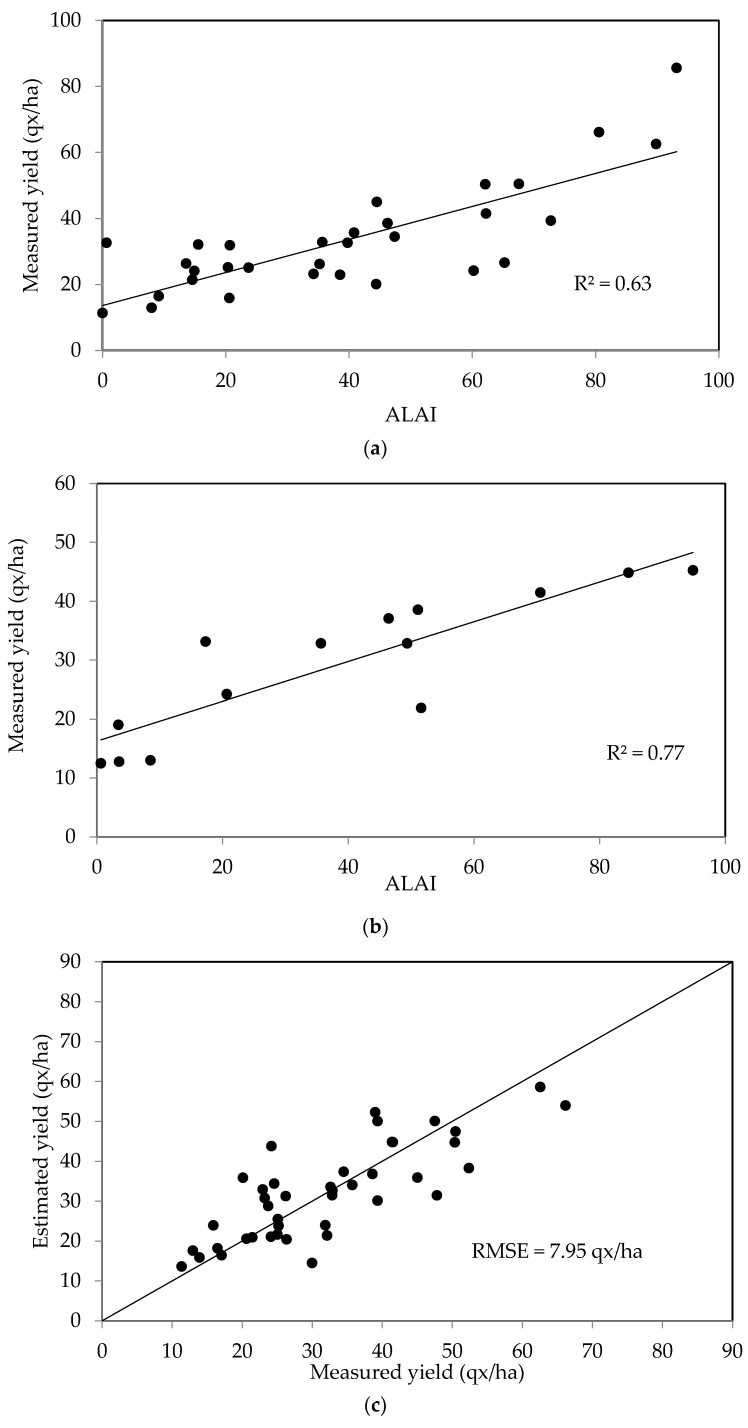

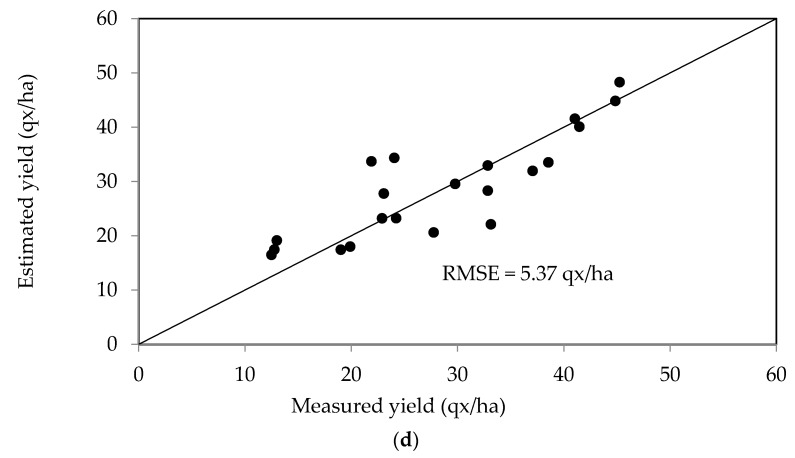
Relationship between the grain yields of wheat (**a**) and barley (**b**), and LAI during the period of maximum growth (A_LAI_). Measured yields shown as a function of the estimated values computed using A_LAI_, the maximum growth LAI, for test plots and two crop years, for wheat (**c**) and barley (**d**).

In a second step, the approach described above was applied to the case of two different cereals: wheat and barley, as shown in Figure 9. The measured and estimated yields are found to be well correlated (R^2^ = 0.63 for wheat, and R^2^ = 0.77 for barley), with (RMSE) errors equal to 7.95 and 5.37 quintals/ha for wheat and barley, respectively.

### 4.3. Yield Estimations for Irrigated and Non-Irrigated Areas

**Figure 10:**
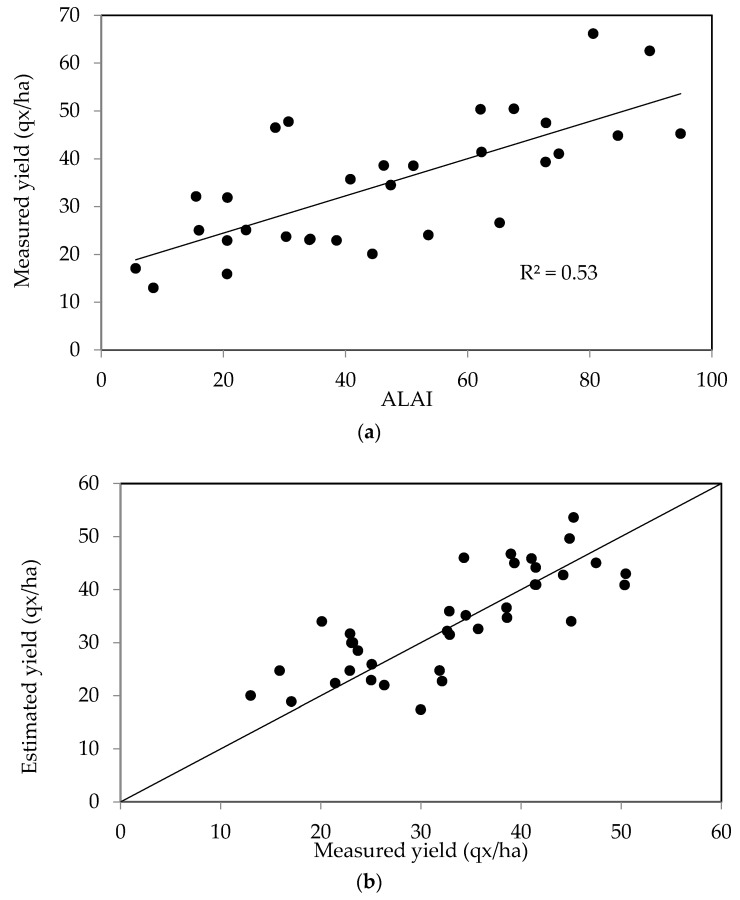
(**a**) Relationship between the grain yields of irrigated cereals and LAI during the period of maximum growth; (**b**) Measured grain yields in irrigated test plots compared with yield estimates, computed using the maximum growth period LAI, over two crop years.

In a third step, we focus on the estimation of grain yields for two types of cereal growing conditions: irrigated and rain-fed, organized according to two separate datasets. The first of these (irrigated cereals) was used to establish a relationship (linear regression, Equation (8)) between the estimated yield and the LAI during the period of maximum growth, as shown in Figure 10a. When the estimated and measured yields are then compared using this regression they are well correlated, with R^2^ equal to 0.53 and the RMSE equal to 6.38 qx/ha (Figure 10b). 

**Figure 11:**
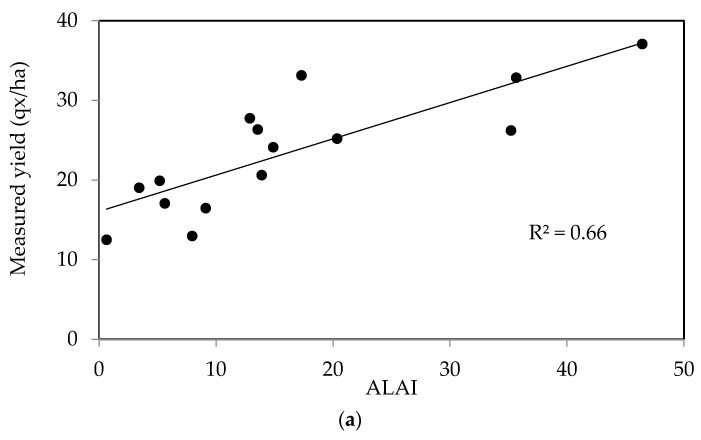

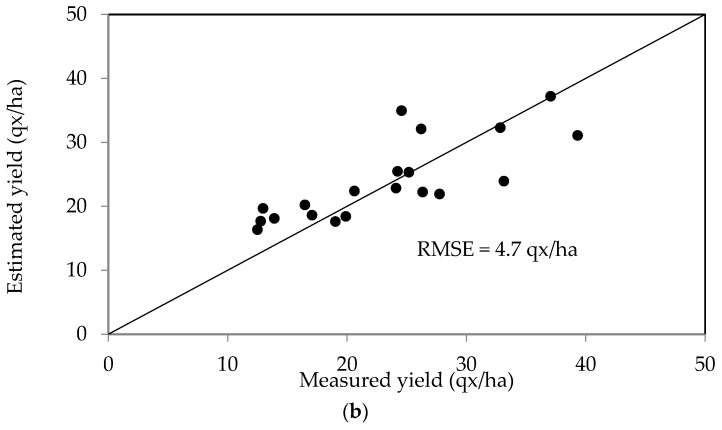
(**a**) Relationship between the grain yields of non-irrigated cereals and LAI during the period of maximum growth; (**b**) Measured yields in non-irrigated test plots, compared with the estimated values computed using the maximum growth LAI over two crop years.

The second dataset corresponding to non-irrigated cereals was analyzed in the same manner, leading to a strong correlation (R^2^_non-irrigated_ = 0.66), with the RMSE equal to 4.7 qx/ha (Figure 11b).

The linear regressions determined for each of these analyses are:

(8) $$\begin{array}{l} GY_{\mathrm{irrigated}}=0.39\times A_{\mathrm{LAI}}+16.68\\ GY_{\mathrm{non}-\mathrm{irrigated}}=0.45\times A_{\mathrm{LAI}}+16.05 \end{array}$$

We note that using this procedure, all of the data points lie inside the second confidence interval, for both types of cereal growing conditions (irrigated and non-irrigated).

### 4.4. Spatialisation of Grain Yield

**Figure 12:**
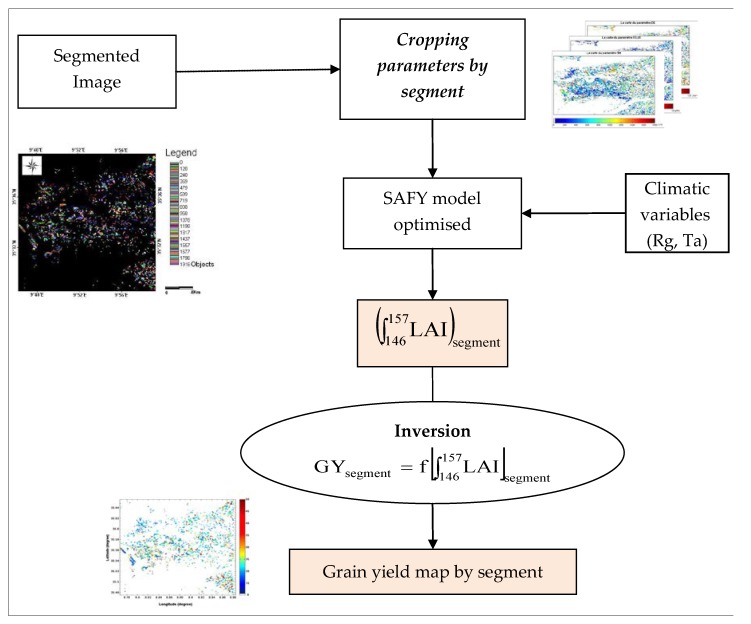
The organigram of the spatialisation of grain-yield.

The spatialisation grain-yield technique is explained in the following organigram (Figure 12). In this section of the study, a distinct inversion was computed for each segment (or plot). This approach is the same as that adopted by [13]. Following calibration of the SAFY model, through the use of multi-temporal LAI satellite maps and LAI values computed for the maximum growth period, yield estimations are proposed for the entire studied site. 

In order to calibrate the SAFY model for each segment, it is first necessary to invert the satellite images of the agricultural year in question, in NDVI maps. Then these images are reversed in LAI via a semi-empirical relationship between NDVI and LAI established for cereal crops and detailed in [13]. The results are LAI maps for each segment and for five different dates.

These maps are used to adjust the simulations performed by the SAFY model. The optimization procedure is applied for each segment in order to minimize the difference between the five observations (LAI derived from SPOT-HRV) and simulated values at observation dates.

**Figure 13:**
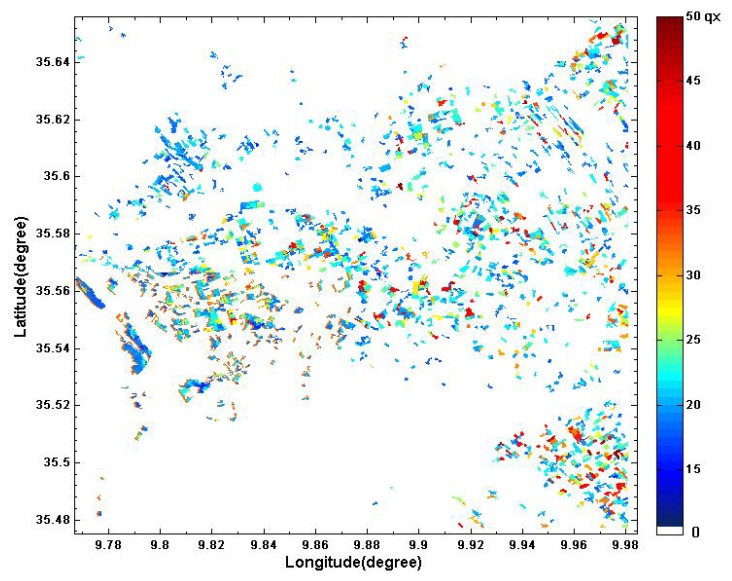
Cereal yield map produced by combining SPOT/HRV multi-temporal acquisitions with SAFY-modeled yields computed with the LAI corresponding to the period of maximum growth.

To estimate the three parameters of the SAFY model (ELUE, D_0_ and S_TT_), the method mentioned in Section 3.2 was applied for each segment. Furthermore, to reduce the effect of local terrain heterogeneities in segment, these three parameters are estimated over cells corresponding to 10 × 10 pixels (approximately 100 m^2^). Maps of the three parameters are generated (ELUE, D_0_, S_TT_). After having generated these parameters, we extract the A_LAI_ variable for each segment. Finally, the empirical relationship determined in Section 4 is used to generate cereal yield maps for each segment (Figure 13).

## 5. Conclusions

The aim of this study was to characterize the dynamics and yields of various cereal plantations in the Kairouan plain, using high-resolution optical data and a simple agricultural model (SAFY). The SAFY model accurately simulates the variation of LAI along the vegetative cycle of cereals.

The estimated grain yield is a function of the LAI during maximum growth (a ten-day period, between the end of March and the beginning of April). For all types of cereal (irrigated or non-irrigated, wheat and barley), a good correlation is observed between the measured and estimated yields. Our validation of the proposed empirical relationships over different types of cover shows that this approach is quite accurate, with an RMSE lower than 7.6 qx/ha. When applied to intensive agriculture on the Kairouan plain, it performs very well, because climatic factors (with global radiation and average daily air temperature used as input parameters), cereal phenological parameters, agro-environmental conditions, and grain growth cycle characteristics (short and narrow, or wide, longer-lasting cycles) as well as senescence, are taken into account. This methodology could be applied in a more operational fashion than the determination of a direct relationship between satellite indices and yield estimations, for two reasons: firstly, optical data may not be usable for periods of many days, due to cloud cover; secondly, since optical measurements are not recorded on the same Julian days each year, they would need to be calibrated with respect to their timing within the 10-day span of the maximum growth period. In future studies, this technique will be tested in an operational context using Sentinel2 data.

## Figures and Tables

**Table 1:** Satellite acquisition dates.

Image	Observation Date	Sensor	θ ^1^
1	24 December 2010	SPOT5	1.75
2	29 January 2011	SPOT5	11.71
3	19 February 2011	SPOT5	5.55
4	17 March 2011	SPOT5	5.50
5	5 April 2011	SPOT4	5.60
6	28 April 2011	SPOT5	8.14
7	18 May 2011	SPOT5	18.23
8	3 July 2011	SPOT4	18.23
9	6 November 2011	SPOT5	5.5
10	13 January 2012	SPOT5	7.78
11	28 February 2012	SPOT5	18.43
12	31 March 2012	SPOT5	7.67
13	4 May 2012	SPOT4	12.7
14	25 May 2012	SPOT4	5.9
15	6 July 2012	SPOT4	7.7

^1^ Incidence angle in degrees.

**Table 2:** Bands characteristics of the satellite SPOT4 and SPOT5.

Mode	Band	Spectral Band
Multispectral	B1: Green	0.50–0.59 µm
	B2: Red	0.61–0.68 µm
	B3: Near Infrared (PIR)	0.79–0.89 µm
	B4: Mid Infrared (MIR)	1.58–1.75 µm

**Table 3:** Range of variation of calibrated parameters for test plots.

Parameter	Unit	Range of Variation
D_0_	Day	15–120
ELUE	g·MJ^−1^	0–10
S_TT_	°C	200–1800
